# Nicotine pouches and youth: emerging patterns and potential cardiovascular risks

**DOI:** 10.3389/fpubh.2025.1632313

**Published:** 2025-11-20

**Authors:** Shaun Abid, Abhinav Aggarwal, Freddy Duarte, Samdish Sethi, Sumedh Iyengar, Stuart Zarich

**Affiliations:** 1 Department of Internal Medicine, Yale New Haven Health, Bridgeport Hospital, Bridgeport, CT, United States; 2 Department of Cardiology, Yale New Haven Health, Bridgeport Hospital, Bridgeport, CT, United States; 3 Department of Cardiology, Houston Methodist Hospital, Houston, TX, United States

**Keywords:** nicotine pouches, cardiovascular risk, adolescents, young adults, nicotine addiction, tobacco harm reduction

## Abstract

The rapid rise in nicotine pouch use among teens and young adults signals a shift in nicotine consumption with significant public health implications. Marketed as discreet and “tobacco free,” these products are often perceived as safer alternatives to smoking, yet their cardiovascular risks remain largely unexamined. Emerging concerns center on the high nicotine content, efficient bloodstream delivery, and growing rates of dual or poly-use with other nicotine products. Adolescents, in particular, face heightened vulnerability due to ongoing physiological development and increased addiction susceptibility. This review explores the potential cardiovascular consequences of nicotine pouch use in youth, emphasizing the urgency of evidence-based research to inform clinical guidance, regulatory action, and prevention strategies.

## 1 Introduction

The landscape of nicotine and tobacco products is undergoing a significant transformation, marked by the emergence and growing market presence of oral nicotine pouches (1, 2). These products represent a critical shift away from traditional combustible cigarettes towards a seemingly “cleaner” form of nicotine delivery (2, 3). Often marketed under the guise of harm reduction and frequently labeled “tobacco free,” nicotine pouches leverage their discrete nature and appealing flavors to capture a growing market share (4–6). However, this surge in popularity, especially within younger demographics, raises profound public health concerns (7, 8). While touted by industry as a less harmful alternative, the actual cardiovascular risks associated with nicotine pouch use remain largely uncharacterized, creating a potential blind spot in public health surveillance and intervention efforts (2, 7).

The core issue lies in the potent pharmacological effects of nicotine itself, coupled with the unique characteristics of pouch delivery systems. Nicotine’s detrimental impact on the cardiovascular system is well documented (9), but pouches introduce nicotine directly into the bloodstream via oral absorption, bypassing pulmonary routes and potentially intensifying systemic effects (10). Furthermore, the nicotine content in many pouches is alarmingly high, often meeting or exceeding levels found in traditional cigarettes, which significantly increases their addictive potential and associated cardiovascular risks (11–13). While exclusive use of oral nicotine pouches may reduce exposure to combustion related toxicants compared with cigarette smoking, dual use maintains chronic nicotine exposure, sympathetic activation, and endothelial dysfunction, thereby potentially diminishing the cardiovascular benefits associated with reduced cigarette consumption. This distinction is particularly relevant in adolescents, whose developing cardiovascular and nervous systems may be more susceptible to nicotine’s hemodynamic and neurochemical effects (7). Exposure during this sensitive period could disrupt normal maturation, leading to heightened addiction susceptibility and potentially predisposing users to early onset hypertension, endothelial dysfunction, and other cardiovascular pathologies (9, 14). The high rates of poly tobacco use where pouches are often used alongside E-cigarettes or other nicotine products may further compound these risks (8, 15). This review aims to synthesize the current understanding of nicotine pouches, focusing specifically on the potential cardiovascular risks they pose to teens and young adults. We will summarize the rise and appeal of these products, review nicotine’s cardiovascular pharmacology, analyze pouch characteristics relevant to risk, evaluate the existing evidence, identify critical knowledge gaps, and discuss the implications for public health policy and prevention. A summary of the key clinical, epidemiological, and cellular studies informing this review is presented in Table 1.

**Table 1:** Summary of clinical, epidemiological, and *in vitro* studies on the cardiovascular risks of nicotine products.

Author (year) [Ref]	Study design	Population	Key findings relevant to cardiovascular risk
Human clinical trials and PK/PD studies			
Mallock-Ohnesorg et al. (2024) (10)	Single-center, 5-arm crossover clinical trial	15 regular cigarette smokers	- 30 mg nicotine pouch led to higher Cmax and AUC than a cigarette. - Use of 20 mg and 30 mg pouches caused significant dose-dependent increases in heart rate and arterial stiffness.
Azzopardi et al. (2022) (23)	Randomized PK study	34 adult tobacco users	- A 4 mg nicotine pouch delivered nicotine comparable to a 4 mg NRT lozenge and more effectively than a 4 mg NRT gum.
Najem et al. (2006) (24)	Clinical study	16 healthy smokers	- NRT (as a proxy for pure nicotine) increases sympathetic nerve activity, heart rate, and blood pressure.
Epidemiological and youth prevalence studies			
Han et al. (2025) (8)	Nationally representative cross-sectional survey (Monitoring the Future)	10,146 U.S. 10th and 12th graders	- Past 30 day nicotine pouch use doubled from 2023 (1.3%) to 2024 (2.6%). - Dual use of pouches and e-cigarettes significantly increased.
Birdsey et al. (2023) (20)	Nationally representative cross-sectional survey (NYTS)	U.S. middle and high school students	- 1.5% (approx. 400,000 students) reported current (past-30-day) nicotine pouch use in 2023.
Meta-analyses of smokeless tobacco (ST)			
Vidyasagaran et al. (2016) (18)	Systematic review and meta-analysis (20 studies)	Adult ST users	- ST use was associated with an increased risk of fatal ischemic heart disease (RR 1.15) and fatal stroke (RR 1.39).
Boffetta and Straif (2009) (28)	Systematic review and meta-analysis	Adult ST users	- ST use was associated with an increased risk of fatal myocardial infarction (RR 1.13) and fatal stroke (RR 1.40).
*In vitro* cellular toxicity studies			
Rinaldi et al. (2023) (17)	In vitro toxicity study	Human gingival fibroblasts (HGF-1)	- Extracts from nicotine pouches induced cytotoxicity. - Upregulated inflammatory (IL6) and oxidative stress (HMOX1) genes, with toxicity not solely dependent on nicotine, implicating flavorings.

AUC, area under the curve; Cmax, maximum concentration; HGF-1, human gingival fibroblasts; HMOX1, Heme Oxygenase 1; IL6, Interleukin-6; NRT, nicotine replacement therapy; NYTS, National Youth Tobacco Survey; PK/PD, pharmacokinetic/pharmacodynamic; Ref, Reference; RR, relative risk; ST, smokeless tobacco.

## 2 Background: the emergence and appeal of nicotine pouches

### 2.1 Product characteristics

Oral nicotine pouches represent a distinct category within the nicotine product market. Defined by their lack of tobacco leaf, these products typically consist of a small, pre portioned pouch containing nicotine (often synthetic or highly purified tobacco derived), fillers (like plant fibers), sweeteners, and various flavorings (1, 12, 16). Users place the pouch between the gum and lip, allowing nicotine absorption through the oral mucosa. Unlike traditional moist snuff, they generally do not require spitting (1). Furthermore, because they do not involve combustion or produce an aerosol cloud like E-cigarettes, nicotine pouches do not generate sidestream emissions, thus posing no secondhand exposure risk to bystanders.

A key concern is the significant variability in their chemical composition. Nicotine content ranges dramatically, from less than 2 mg to nearly 50 mg per pouch in some analyses. The pH of these products is also highly variable (ranging from 5.5 to over 10) and typically alkaline (median pH 8.8) (12, 13). This high pH increases the proportion of unprotonated (“free base”) nicotine (median 86%), which is more readily absorbed across oral membranes, potentially increasing the speed of delivery and the addictive potential (10, 12, 13). Analyses have shown that some pouches contain free-base nicotine proportions exceeding 95% and, despite being marketed as “tobacco free,” also harbor additional concerning chemicals from ubiquitous flavorings to cytotoxic agents like cinnamaldehyde and eugenol (12, 17). Furthermore, carcinogenic tobacco specific nitrosamines (TSNAs), such as N-Nitrosonornicotine and 4-(Methylnitrosamino)-1-(3-pyridyl)-1-butanone, have been detected in many nicotine pouch products, albeit generally at much lower levels than in traditional smokeless tobacco or cigarettes (13, 18).

### 2.2 Market landscape and growth

The emergence of nicotine pouches is largely driven by major tobacco companies, including British American Tobacco (Velo, Lyft), Swedish Match/Philip Morris International (Zyn), Altria (On!), and others, who introduced these products to the US and European markets starting around 2016 (1, 4). Since their introduction, sales have increased exponentially (2, 4). Zyn rapidly became the market leader in the US, although Velo has invested heavily in advertising (4). The regulatory landscape is evolving, and in the United States, products containing nicotine from either tobacco derived or synthetic sources now fall under FDA authority (7). Manufacturers must submit Premarket Tobacco Product Applications. In a significant move, the FDA authorized the marketing of 20 Zyn products in early 2025, deeming them “appropriate for the protection of public health” based on lower toxicant levels compared to cigarettes and evidence supporting complete switching among some adult smokers, while imposing strict marketing restrictions to limit youth appeal (19).

### 2.3 Prevalence and use patterns (focus on youth/young adults)

**Figure 1:**
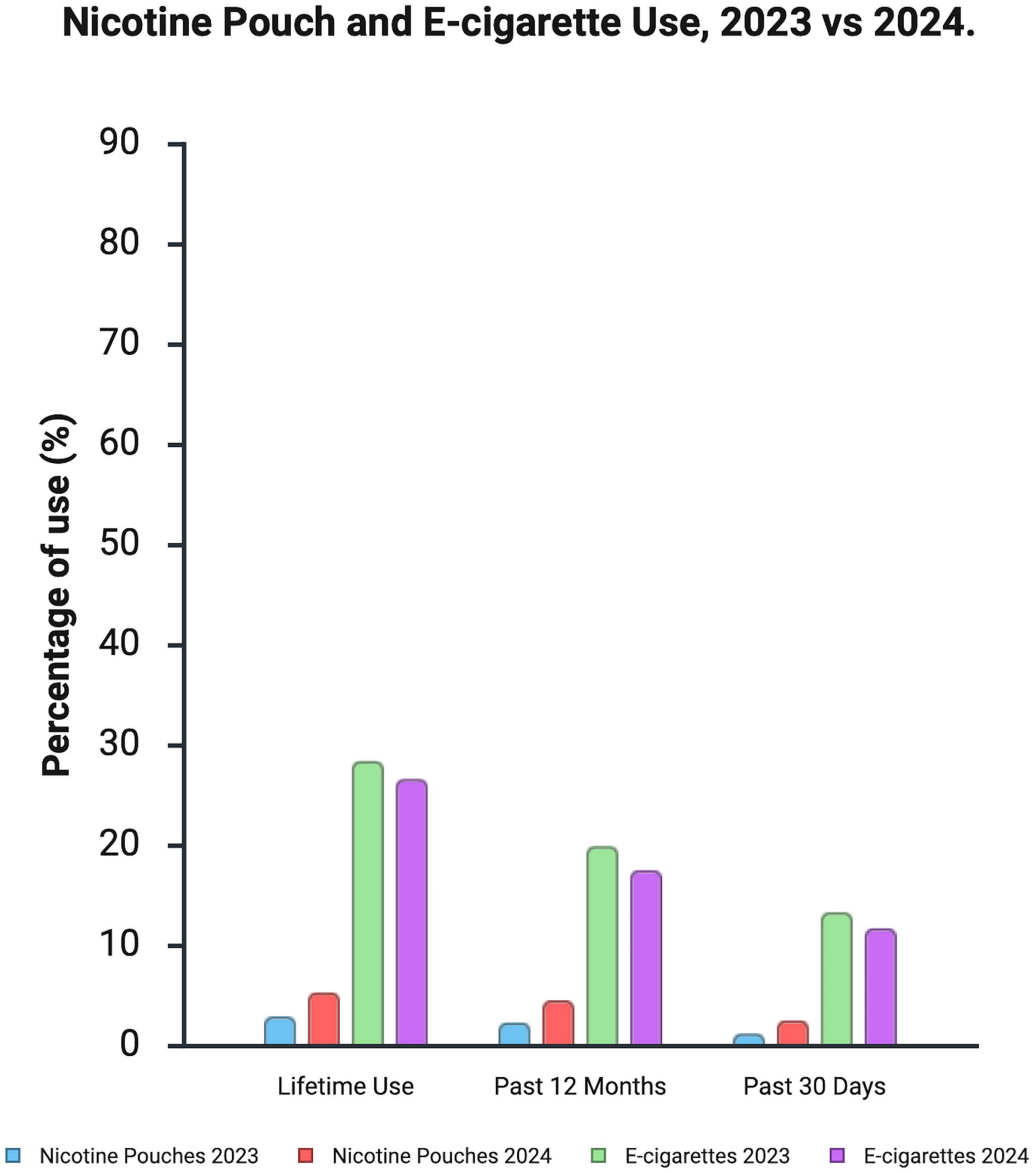
Nicotine pouch and E-cigarette use among 10th and 12th graders in 2023 vs. 2024. Data from the National Youth Tobacco Survey demonstrating the increase in past 30 day, past 12 month, and lifetime use for both product categories.

Nicotine pouch use has shown a concerning upward trend among adolescents and young adults. The 2023 National Youth Tobacco Survey found that 1.5% of US middle and high school students (approximately 400,000) reported current (past 30 day) use, making it more prevalent than traditional smokeless tobacco among this group (20). Data from the Monitoring the Future study showed significant increases in past 30 day use among 10th and 12th graders between 2023 and 2024, rising from 1.3 to 2.6% (risk difference 1.3, 95% CI: 0.5–2.1; *p* < 0.001), indicating a doubling in use in just 1 year (8). Figure 1 illustrates the significant increase in nicotine pouch use, in contrast to the slight decrease observed in E-cigarette use, among this demographic.

While overall prevalence remains lower than E-cigarettes, the rapid growth is notable (2, 8, 20). Use is higher among males, non Hispanic White youth, those residing in rural areas, older adolescents (12th vs. 10th grade), and those without plans for 4 year college (8, 21). Co-use with other products, particularly E-cigarettes, is also increasing among youth, a pattern also seen in adult E-cigarette users who may use pouches situationally (8, 15). Susceptibility among tobacco naive youth is estimated between 9 and 21% (2).

### 2.4 Marketing strategies and youth appeal

Marketing efforts for nicotine pouches employ strategies known to appeal to younger demographics. Flavors are central to their appeal and marketing, with fruit, mint/menthol, and wintergreen being prominent. Flavor is cited as a primary reason for initiation, especially among young adults (18–24) and never smokers (4, 6). Marketing campaigns heavily emphasize convenience and discretion, using slogans like “use anywhere,” “smoke free,” “spit free,” and highlighting “freedom.” The ‘smoke free’ and ‘use anywhere’ slogans, in particular, directly capitalize on the product’s lack of combustion and secondhand emissions, framing it as a modern alternative that bypasses the social and legal restrictions placed on smoking and vaping. The “tobacco free” descriptor is frequently used, potentially contributing to reduced harm perceptions. Advertising occurs across diverse platforms, including radio, television, print, online displays, direct mail, and significantly, social media channels often frequented by youth, sometimes involving influencers. Entertainment, sports, and news/weather channels are common platforms. These tactics raise concerns about undermining tobacco control efforts and attracting youth to nicotine (2, 4, 6).

## 3 Nicotine pharmacology and cardiovascular effects

### 3.1 Mechanisms of nicotine action

As detailed by Benowitz (22) in his comprehensive review of nicotine pharmacology, nicotine’s addictive potential stems from its complex interaction with the nervous system, involving rapid central nervous system penetration and subsequent activation of neural reward pathways. Nicotine exerts its powerful effects primarily by binding to and activating nicotinic acetylcholine receptors (nAChRs) located throughout the central and peripheral nervous systems. This binding triggers the release of various neurotransmitters. Critically for addiction, nicotine stimulates dopamine release in the brain’s reward pathways (mesolimbic system), signaling pleasure and reinforcing drug taking behavior. It also increases levels of norepinephrine and epinephrine via sympathetic nervous system activation. Other neurotransmitters like glutamate and GABA are also modulated, contributing to the complex effects on mood, arousal, and cognition. Chronic exposure leads to neuroadaptations, including nAChR upregulation and desensitization, which are thought to underlie tolerance and withdrawal symptoms upon cessation. Withdrawal is characterized by negative affective states (irritability, anxiety, depression, anhedonia) and craving, partly mediated by dopamine deficits and activation of stress pathways involving corticotropin releasing factor. Conditioned cues associated with nicotine use become powerful triggers for craving and relapse.

### 3.2 Nicotine delivery from pouches

Pharmacokinetic studies demonstrate that nicotine pouches can be highly efficient nicotine delivery systems. Absorption occurs via the oral mucosa, bypassing first pass metabolism (10). The rate and extent of absorption are significantly influenced by product characteristics, particularly pH and the resulting proportion of free base nicotine. Studies show wide variability in nicotine delivery across different pouch products (10, 12). Some low dose pouches (e.g., 4 mg) deliver nicotine comparably to nicotine replacement therapy (NRT) lozenges and more effectively than NRT gum (23). However, high dose pouches (e.g., 30 mg) can achieve peak plasma concentrations and total nicotine exposure that meet or even significantly exceed those typically seen after smoking a single cigarette. Furthermore, the initial rate of nicotine rise from some high-dose pouches can be as rapid as that from cigarettes, suggesting a potential for dependence similar to other fast delivery nicotine systems (10, 12, 23). Extraction rates also vary, meaning the labeled dose does not always predict the delivered dose.

While pharmacokinetic studies demonstrate efficient nicotine delivery, definitive comparative data on dependence potential between nicotine pouches, cigarettes, and E-cigarettes remain limited. Emerging evidence indicates that rapid nicotine absorption may increase reinforcing effects, but systematic studies quantifying addictive potential are lacking (15). Additional research is needed to assess long-term patterns of use, dependence trajectories, and cessation outcomes across different nicotine-delivery modalities.

### 3.3 Established acute cardiovascular effects of nicotine

Nicotine’s primary acute cardiovascular effects stem from its activation of the sympathetic nervous system. This leads to the release of catecholamines (epinephrine, norepinephrine), resulting in immediate increases in heart rate, blood pressure, and myocardial contractility, thereby increasing cardiac workload and oxygen demand (24). These effects have been corroborated in both ambulatory monitoring studies showing elevated daytime blood pressures in smokers, and experimental studies demonstrating nicotine induced endothelial dysfunction and altered vascular reactivity (25, 26). Nicotine also causes vasoconstriction in certain vascular beds, including coronary arteries, which can potentially impair myocardial blood flow, particularly in individuals with underlying disease (9). Studies using NRT have confirmed that nicotine itself increases sympathetic nerve activity and elevates heart rate and blood pressure, effects that persist even during physiological stress like hypoxia (24). Nicotine can also acutely impair endothelial function, reducing nitric oxide availability and potentially contributing to oxidative stress and inflammation (9, 27, 28). Furthermore, nicotine can lower the threshold for cardiac arrhythmias (9). Studies specifically examining nicotine pouches confirm these effects, showing dose dependent increases in heart rate, blood pressure, and markers of arterial stiffness shortly after use (10).

### 3.4 Potential long term cardiovascular consequences of nicotine exposure

While the acute effects are clear, the long term cardiovascular consequences of sustained nicotine exposure (distinct from smoking) are less certain but concerning. Chronic sympathetic activation can contribute to cardiac remodeling, hypertension development, and increased risk of arrhythmias (9). Nicotine’s potential to induce endothelial dysfunction, oxidative stress, and inflammation suggests a plausible role in accelerating atherogenesis (9, 27). It also adversely affects lipid profiles (lowering HDL, potentially increasing LDL via lipolysis) and promotes insulin resistance, both established cardiovascular risk factors (9, 22). While epidemiological studies on traditional smokeless tobacco users show lower overall CVD risk compared to smokers, they indicate an increased risk of *fatal* myocardial infarction and stroke (18, 29). This suggests nicotine exposure may be particularly dangerous in the context of existing cardiovascular disease or acute ischemic events (7). Given that some nicotine pouches deliver nicotine levels comparable to or higher than traditional ST or cigarettes, these long term risks associated with chronic nicotine exposure are highly relevant to pouch users, especially those initiating use in adolescence (7, 9, 10, 12).

## 4 Unique vulnerabilities of teens and young adults

### 4.1 The developing adolescent brain and cardiovascular system

Adolescence is a critical neurodevelopmental period characterized by significant remodeling of brain circuits, particularly those involving the prefrontal cortex and limbic system, which govern executive function, reward processing, and emotional regulation. Nicotinic receptors play a crucial role in modulating this maturation process. Consequently, the adolescent brain exhibits unique sensitivity to nicotine (14). Nicotine exposure during this time disrupts normal developmental trajectories, altering neurotransmitter systems (especially dopamine and serotonin) and leading to lasting changes in brain structure and function (14, 22). This disruption manifests as altered behavioral responses to nicotine compared to adults—including enhanced reward sensitivity, reduced aversion, and blunted withdrawal symptoms—creating a window of heightened vulnerability to addiction. These nicotine induced changes can persist into adulthood, potentially causing long term deficits in cognitive functions like attention and impulse control, and increasing susceptibility to anxiety and depression (14). Although direct evidence on nicotine’s effects in the developing cardiovascular system is limited, its well established impact in adults particularly through sustained sympathetic activation and endothelial dysfunction raises credible concerns that early exposure during adolescence may disrupt normal cardiovascular maturation and contribute to the early development of risk factors for cardiovascular disease (9).

### 4.2 Factors driving youth initiation and use

Multiple factors contribute to the initiation and continued use of nicotine pouches among teens and young adults. Among them, the availability of appealing flavors is a key driver frequently cited as a primary reason for both starting and sustaining use particularly among young users (4, 6, 8). While experimental evidence also supports nicotine’s reinforcing properties in conditioned behavior, flavors enhance product appeal and reinforce use patterns (22). Marketing strategies emphasizing discretion (“use anywhere”) and convenience resonate with this age group (4, 6). Peer influence and the normalization of vaping behaviors may also play a role (8). Furthermore, misperceptions about harm are prevalent. Many youth view pouches, often marketed as “tobacco free,” as significantly less harmful than cigarettes or even E-cigarettes. As a result, they may underestimate the risks associated with nicotine itself (2). Susceptibility measures like curiosity and willingness to try are high among youth, even non users, and are strongly associated with subsequent initiation (2, 30, 31).

### 4.3 Poly tobacco use

A concerning trend among adolescent nicotine pouch users is the high rate of poly tobacco use, particularly concurrent use with E-cigarettes (8). Studies suggest that initiating one tobacco product, like E-cigarettes or potentially pouches, increases susceptibility to and lowers harm perceptions of other tobacco products (30). This “catalyst” effect, supported by findings that initial E-cigarette use predicts future smoking and that product initiation correlates with increased susceptibility to other products, means that pouch use might not only establish nicotine dependence but could also serve as a gateway to initiating or maintaining the use of other, potentially more harmful, products like cigarettes or E-cigarettes (30, 31). Simultaneously using several nicotine containing items may intensify nicotine addiction and can worsen related health problems, including those affecting the heart and blood vessels. This could be a result of greater total nicotine intake and contact with a wider array of harmful substances from the different products used.

## 5 Assessing the cardiovascular threat of nicotine pouches in youth

### 5.1 Direct evidence (limited)

Direct evidence on the cardiovascular effects of nicotine pouches, especially in young people, is scarce. However, available studies provide concerning initial data. Clinical research demonstrates that high dose nicotine pouches can elicit acute cardiovascular responses (increased heart rate, blood pressure, arterial stiffness) comparable in magnitude to smoking a cigarette (10). *In vitro* studies using extracts from nicotine pouches have shown potential cellular toxicity, including cytotoxic effects and the activation of inflammatory and oxidative stress pathways in human gingival fibroblasts (17). While direct evidence linking these specific pouch effects to cardiovascular outcomes is limited in these studies, such cellular pathways (inflammation, oxidative stress) are recognized contributors to cardiovascular pathology, a link extensively discussed for other nicotine products like E-cigarettes (27).

### 5.2 Extrapolated evidence

Given the limited direct evidence, assessing the potential cardiovascular threat relies heavily on extrapolating from known nicotine pharmacology and data from related products. The established cardiovascular risks of nicotine itself are directly applicable, particularly considering the high and rapidly delivered nicotine doses achievable with some pouches (7, 9, 10, 23). Lessons from decades of research on traditional ST are also relevant (7). While ST products differ in composition (containing tobacco leaf and higher levels of TSNAs), they share the oral route of nicotine absorption and have been linked, particularly in recent comprehensive reviews, to increased risk of fatal cardiovascular events (MI and stroke) (18, 29). Although non fatal risks appear lower, especially for low TSNA Swedish snus compared to products used in Asia or the US, the association with fatal events underscores the potential danger of chronic oral nicotine exposure to the cardiovascular system (7, 18). The combination of high nicotine concentrations delivered efficiently to a physiologically vulnerable adolescent population raises significant concern for both acute events (in susceptible individuals) and the long term development of cardiovascular disease (10, 12–14).

### 5.3 The addiction factor

The high addictive potential of nicotine pouches is a crucial factor amplifying cardiovascular risk (10, 22). Products delivering high nicotine doses rapidly, similar to cigarettes, are more likely to establish strong dependence (10, 12). This dependence drives sustained exposure to nicotine’s harmful cardiovascular effects and makes cessation more difficult, prolonging the period of elevated risk. While early generation E-cigarettes were associated with lower dependence than cigarettes, this may not apply to modern high nicotine pouches, which deliver nicotine efficiently (10, 15).

## 6 Knowledge gaps and future research priorities

Despite growing use, significant knowledge gaps remain regarding the cardiovascular impact of nicotine pouches, particularly in youth. There is an urgent need for longitudinal studies tracking cardiovascular health indicators (e.g., blood pressure, arterial stiffness, endothelial function biomarkers, subclinical atherosclerosis) and clinical events (MI, stroke) in adolescents and young adults who initiate and continue using nicotine pouches, comparing them to non users and users of other tobacco products. Research must investigate the specific cardiovascular toxicity of non nicotine constituents, especially common flavor chemicals, both individually and in mixtures, as *in vitro* data suggest they contribute to adverse effects (17). Direct comparative studies are needed to rigorously assess the relative cardiovascular risk profile of nicotine pouches versus cigarettes, E-cigarettes, and traditional smokeless tobacco, using both biomarker and clinical endpoints. Crucially, research must focus on the impact of pouch use on the developing cardiovascular system during adolescence, utilizing appropriate preclinical models and sensitive clinical measures. Finally, understanding how nicotine pouches fit into tobacco use patterns is vital. Key questions remain unanswered. Do they function primarily as a cessation aid? Are they a bridge to other tobacco products? Do they help maintain nicotine dependence during periods when smoking is restricted? Or are they an initiation product for nicotine naive youth? Addressing these questions is essential to guide evidence based public health strategies.

## 7 Public health implications and policy recommendations

The rise of nicotine pouches necessitates a multipronged public health response. Continued surveillance of use patterns through national surveys (NYTS, PATH, MTF) is critical to monitor trends, identify vulnerable subgroups, and track poly use (8, 15, 20). Robust regulation is paramount. This includes enforcing restrictions on marketing tactics that appeal to youth, such as banning all non tobacco flavors (including mint/menthol and coolants), restricting online sales and social media promotion, and prohibiting misleading “tobacco free” or implicit health claims (4, 7). Establishing product standards is crucial, including setting maximum limits on nicotine concentration and regulating pH to reduce free base nicotine levels and abuse liability, requiring full disclosure of ingredients, and mandating standardized, clear labeling of nicotine content per pouch (7, 10, 12, 13). Taxation policies should be applied equitably across all nicotine products to discourage initiation and use. Strengthening and enforcing age verification for sales is essential (7). Prevention and education efforts must include public awareness campaigns specifically addressing the risks of nicotine pouches for youth and young adults, countering industry harm reduction narratives that may inadvertently promote initiation (2). Educating parents, teachers, and healthcare providers is also key. From a clinical perspective, healthcare professionals should routinely screen adolescents and young adults for use of *all* nicotine products, including pouches, provide counseling on the risks, and offer evidence based cessation support. While pouches might be considered only for adult smokers who have failed FDA approved cessation methods, the goal must remain complete cessation of all nicotine products (7).

## 8 Conclusion

The rapid proliferation and increasing use of oral nicotine pouches, especially among teens and young adults, represent a significant and emerging public health challenge. Marketed with appealing flavors and perceived as a less harmful, discreet alternative, these products deliver substantial, rapidly absorbed doses of nicotine, posing a considerable addiction risk and a plausible threat to cardiovascular health. While direct long term data are lacking, the known adverse cardiovascular pharmacology of nicotine, combined with the high exposure potential from pouches and the unique vulnerability of the developing adolescent cardiovascular and nervous systems, creates a strong basis for concern. Critical knowledge gaps regarding the long term cardiovascular consequences in young users must be addressed urgently through rigorous longitudinal research. Proactive, evidence informed public health strategies encompassing robust regulation (especially concerning flavors, nicotine levels, and marketing), targeted prevention campaigns, and clinical screening and cessation support are essential to mitigate the potential cardiovascular burden threatened by these novel nicotine products on the next generation.
